# Understanding How Physical Exercise Improves Alzheimer’s Disease: Cholinergic and Monoaminergic Systems

**DOI:** 10.3389/fnagi.2022.869507

**Published:** 2022-05-18

**Authors:** Boyi Zong, Fengzhi Yu, Xiaoyou Zhang, Wenrui Zhao, Peng Sun, Shichang Li, Lin Li

**Affiliations:** ^1^Key Laboratory of Adolescent Health Assessment and Exercise Intervention of Ministry of Education, East China Normal University, Shanghai, China; ^2^College of Physical Education and Health, East China Normal University, Shanghai, China

**Keywords:** Alzheimer’s disease, exercise, G protein-coupled receptor, acetylcholine, norepinephrine, serotonin, dopamine

## Abstract

Alzheimer’s disease (AD) is an age-related neurodegenerative disorder, characterized by the accumulation of proteinaceous aggregates and neurofibrillary lesions composed of β-amyloid (Aβ) peptide and hyperphosphorylated microtubule-associated protein tau, respectively. It has long been known that dysregulation of cholinergic and monoaminergic (i.e., dopaminergic, serotoninergic, and noradrenergic) systems is involved in the pathogenesis of AD. Abnormalities in neuronal activity, neurotransmitter signaling input, and receptor function exaggerate Aβ deposition and tau hyperphosphorylation. Maintenance of normal neurotransmission is essential to halt AD progression. Most neurotransmitters and neurotransmitter-related drugs modulate the pathology of AD and improve cognitive function through G protein-coupled receptors (GPCRs). Exercise therapies provide an important alternative or adjunctive intervention for AD. Cumulative evidence indicates that exercise can prevent multiple pathological features found in AD and improve cognitive function through delaying the degeneration of cholinergic and monoaminergic neurons; increasing levels of acetylcholine, norepinephrine, serotonin, and dopamine; and modulating the activity of certain neurotransmitter-related GPCRs. Emerging insights into the mechanistic links among exercise, the neurotransmitter system, and AD highlight the potential of this intervention as a therapeutic approach for AD.

## Introduction

With a rapidly aging global population, the number of individuals living with dementia has more than doubled from 1990 to 2016 (Collaborators, 2019). Currently, dementia affects more than 50 million people worldwide, and this number is projected to reach 152 million by 2050 (Patterson, 2020). Alzheimer’s disease (AD) is the most common cause of dementia No authors listed (2021). The earliest and most prominent symptom of AD is memory decline; however, as the disease progresses, it can also cause a large number of psychological and behavioral changes, which create immense distress for patients and care givers (Geda et al., 2013; Ismail et al., 2022). Although AD represents a growing burden for families and society, its complexity and multifactorial etiology pose unique challenges in the study of its pathogenesis and the development therapies.

Based on previous studies, extracellular senile plaques composed of deposits of β-amyloid (Aβ) peptide and neurofibrillary tangles (NFTs) of hyperphosphorylated tau protein are widely recognized as pathological hallmarks of AD (Jack et al., 2013; Chong et al., 2021). However, despite great expectations, only a small number of antibodies targeting Aβ or tau have been selected for investigation in clinical trials (Long and Holtzman, 2019). In June 2021, aducanumab (aducanumab-avwa; Aduhelm™), a human immunoglobulin gamma 1 monoclonal antibody directed against aggregated soluble and insoluble forms of Aβ, was approved by the Food and Drug Administration (FDA) as the first immunotherapy for AD (Dhillon, 2021). As aducanumab is a new drug, its efficacy, durability, and side effects still need to be further evaluated (Day et al., 2022). The neurotransmitter system is among the earliest affected and most strongly affected systems during the development of AD. Accumulating evidence shows that aberrant neurotransmission, especially in the cholinergic system, is a major pathological factor in AD (Hampel et al., 2018; Richter et al., 2018). In addition to aducanumab and memantine, another three drugs for AD treatment have been approved by the FDA, namely donepezil, galantamine, and rivastigmine. These are all inhibitors of the enzyme acetylcholinesterase (AChE), which can effectively increase acetylcholine (ACh) levels and offer some symptomatic benefit for patients with AD (Joe and Ringman, 2019). Furthermore, AD is closely associated with impaired monoaminergic neurotransmission, mainly involving the dopaminergic, serotoninergic, and noradrenergic systems (Simic et al., 2017; Morgese and Trabace, 2019). In addition to AD, defects in the cholinergic or/and monoaminergic neurotransmitter systems have been shown to be associated with pathological development and clinical manifestations of primary tauopathies, including frontotemporal dementia, progressive supranuclear palsy, and corticobasal syndrome (Huey et al., 2006; Murley and Rowe, 2018), as well as tauopathies with environmental exposure such as chronic traumatic encephalopathy (Mufson et al., 2021) and Parkinsonism-dementia complex of Guam (Nakano and Hirano, 1983; Yamamoto and Hirano, 1985; Goto et al., 1990). Thus, enzymes and proteins involved in the anabolism and catabolism of neurotransmitters and their receptors are potential therapeutic targets for multiple tauopathies including AD.

**FIGURE 1:**
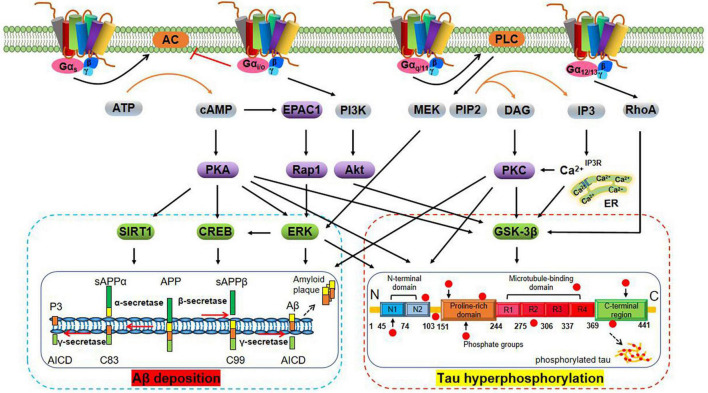
Schematic representation of G protein regulation of Aβ deposition and tau hyperphosphorylation-related signaling pathways. Aβ is derived from the amyloidogenic cleavage of the transmembrane amyloid precursor protein (APP) mediated by α-, β-, and γ-secretases. In the amyloidogenic pathway, APP is first cleaved by the β-secretase (BACE-1), which generates soluble amyloid precursor protein β (sAPPβ) and the β-C-terminal fragment (β-CTF, also termed C99). The latter is cleaved by γ-secretase to generate the Aβ peptide and the amyloid intracellular domain (AICD). The Aβ peptide aggregates to form Aβ oligomers (oAβ) and extracellular amyloid plaques (Hamm et al., 2017). In the non-amyloidogenic pathway, cleavage of APP by α-secretases [especially A disintegrin and metalloprotease 9 (ADAM9), ADAM10, and ADAM17] generates sAPPα and carboxy-terminal fragment termed C83. Subsequent cleavage of C83 by the γ-secretase complex yields the AICD and a short fragment termed P3 (De Strooper, 2010). Tau is an axonal protein expressed in mature neurons that promotes the self-assembly of tubulin into microtubules and its stabilization. The physiological function of tau depends on its phosphorylation status and is regulated by tau protein kinase and phosphatase. In AD brains, tau hyperphosphorylation under the abnormal regulation of protein kinases [e.g., glycogen synthase kinase-3β (GSK-3β)] results in the formation of NFTs (Congdon and Sigurdsson, 2018). Activation of Gα_s_ protein activates adenylyl cyclase (AC) and promotes cyclic adenosine monophosphate (cAMP) generation. cAMP regulates Aβ deposition and tau hyperphosphorylation via activation of the extracellular regulated protein kinase (ERK) (Angulo et al., 2003), cAMP-response element binding protein (CREB) (Wang Z. et al., 2018), silent mating type information regulation 2 homolog 1 (SIRT1), and GSK3β interaction protein/GSK3 (Ko et al., 2019; Zhang Z. et al., 2020) signaling pathways in a protein kinase A (PKA)-dependent manner (Lebel et al., 2009), and exchange protein directly activated by cAMP 1 (EPAC1)/Rap1 in a PKA-independent manner (Maillet et al., 2003). By contrast, activation of Gα_i/o_ protein inhibits the cAMP/PKA pathway. Activation of Gβγ protein may regulate tau phosphorylation through phosphatidylinositol 3-kinase (PI3K)/protein kinase B (PKB, also termed Akt)/GSK-3β pathway (Wang et al., 2016). Activation of Gα_q/11_ protein activates phospholipase C (PLC) to produce inositol trisphosphate (IP3) and diacylglycerol (DAG), which in turn increases concentrations of intracellular calcium (Ca^2+^) and activates PKC, and leads to blocking of tau hyperphosphorylation and inactivation of GSK-3β (Medeiros et al., 2011; Garwain et al., 2020). Furthermore, the Gα_q/11_/PLC pathway can regulate Aβ generation through the MEK/ERK/CREB pathway, among others (Wang Z. et al., 2018). Activation of Gα_12/13_ protein activates GSK-3β in a manner dependent on Ras homolog gene family, member A (RhoA) (Sayas et al., 2002).

A wide variety of molecular structures have been found to act as neurotransmitter receptors, the most numerous of which are ligand-gated channels and G protein-coupled receptors (GPCRs) (Nicoll et al., 1990). GPCRs, a large superfamily of receptors with seven transmembrane segments, are the targets of approximately 34% of all drugs approved by the FDA for the treatment of various diseases (Hauser et al., 2017). GPCRs detect and translate extracellular events such as changes in neurotransmitter concentration into intracellular responses by activating signaling effector proteins, i.e., heterotrimeric G proteins, GPCR kinases (GRKs), and arrestins (Wang W. J. et al., 2018). Heterotrimeric G proteins are key transducers of GPCRs and have alpha (α), beta (β), and gamma (γ) subunits. The β and γ subunits remain associated throughout the signaling cycle and form the Gβγ dimer. Gα proteins can be divided into four main classes according to their Gα sequence: Gα_s_, Gα_i/o_, Gα_q/11_, and Gα_12/13_ (Simon et al., 1991). A large body of evidence indicates that G protein-mediated signaling pathways have important regulatory roles in Aβ deposition and tau hyperphosphorylation (see Figure 1 for details). Moreover, the roles of GRKs including GRK5 (Zhao J. et al., 2019) and arrestins such as β1-arrestin (Liu et al., 2013) and β2-arrestin (Thathiah et al., 2013), among others, should not be ignored.

Extensive studies have investigated the effects of physical activity or exercise on individuals with AD. Prior cross-sectional studies confirmed that levels of physical activity or exercise in autosomal dominant AD mutation carriers were associated with levels of AD biomarkers within the central nervous system and with cognitive performance (Brown et al., 2017; Muller et al., 2018). Furthermore, longitudinal studies demonstrated that low levels of physical activity were associated with a higher risk of dementia in older individuals (Tan et al., 2017), whereas regular physical activity could reduce the risk or delay the onset of dementia and AD, especially among genetically susceptible individuals (Rovio et al., 2005). Nowadays, physical activity and exercise have been widely acknowledged as effective strategies for improving AD pathology and AD-associated cognitive impairment (Northey et al., 2018; Jia et al., 2019; de Farias et al., 2021). From a mechanistic perspective, macroscopically, regular exercise has been shown to alleviate some abnormalities of brain structure and function and to increase cerebral blood flow in subjects with mild cognitive impairment (MCI) and AD (Broadhouse et al., 2020; Tomoto et al., 2021; Yu et al., 2021); microscopically, exercise training not only increases levels of exerkines (e.g., irisin, Lourenco et al., 2019; Islam et al., 2021) and metabolic factors (e.g., lactate, El Hayek et al., 2019) in the peripheral circulation, which act on the AD brain indirectly, but also exert direct neuroprotective effects by increasing levels of brain-derived neurotrophic factor (BDNF) (Wang and Holsinger, 2018) and promoting adult hippocampal neurogenesis (Choi et al., 2018), enhancing synaptic plasticity (Mu et al., 2022), reducing neuroinflammation and oxidative stress (Zhang et al., 2019), and ameliorating Aβ deposition and tau hyperphosphorylation (Brown et al., 2019). Strikingly, the activity of central neurotransmitter systems seems to be strongly modulated by exercise. Changes in physiological levels of neurotransmitters and activity of GPCRs may represent important pathways by which exercise improves AD. This article summarizes the correlations between abnormalities in the cholinergic and monoaminergic systems and the development of AD neuropathology, as well as the underlying mechanisms by which exercise affects these processes.

## Cholinergic System

### Cholinergic Disturbances in Alzheimer’s Disease

The basal forebrain complex comprising the medial septum, horizontal and vertical diagonal band (VDB) of Broca and nucleus basalis of Meynert (NBM), is an important structural basis for cholinergic projections (Schliebs and Arendt, 2011). Aging leads to moderate degenerative changes in basal forebrain cholinergic neurons (BFCNs) located in this complex, accompanied by loss of the neurotransmitter ACh, resulting in reduction of cholinergic projections in the cerebral cortex and hippocampus (Schliebs and Arendt, 2011; Ruan et al., 2018). Considerable advances have been made in our understanding of the roles of the cholinergic system in the development of AD since the 1970s and 1980s (Bowen et al., 1976; Davies and Maloney, 1976; Whitehouse et al., 1982; Coyle et al., 1983). Abnormal cholinergic activity and function have been extensively observed in both AD animal models (Zhu et al., 2017; Xhima et al., 2020) and human patients (Fernandez-Cabello et al., 2020; Machado et al., 2020). Notably, the loss of cholinergic neurotransmission in AD is mainly due to the dysfunction of BFCNs and a decrease in physiological levels of ACh at the cholinergic synapse. The degeneration of BFCNs in the NBM and VDB induced by Aβ deposition and tau hyperphosphorylation is an important pathological mechanism underlying cognitive deficits in AD patients (Vana et al., 2011; Baker-Nigh et al., 2015; Gonzalez et al., 2021). Thus, there is an urgent need for therapeutics and delivery methods that slow or reverse the degeneration of cholinergic neurons in AD.

ACh, an important neurotransmitter in cholinergic transmission, participates in a range of cognitive activities including attention, learning, and memory (Hasselmo, 2006; Haam and Yakel, 2017). The cholinergic hypothesis of AD has inspired research into the roles of ACh in this disease, resulting in a widely held view that restoring levels of ACh may be useful in treating AD. ACh can promote the soluble Aβ peptide conformation rather than the aggregation-prone β-sheet conformation (Grimaldi et al., 2016; Polverino et al., 2018), and regulate tau phosphorylation (Rubio et al., 2006), to combat Aβ and tau pathology. Of concern, the synthesis, transport, release, and metabolism of ACh are multi-step processes that need to be finely modulated by choline, acetyl coenzyme A, choline acetyltransferase (ChAT), vesicular acetylcholine transporter, AChE, and choline transporters, among others (Ferreira-Vieira et al., 2016). However, the progressive dysfunction of certain key components during aging results in decreased ACh levels and subsequent memory deficits (Bartus et al., 1982). Currently, much attention is paid to changes in the activity of ACh-synthesizing enzyme ChAT and ACh-degrading enzyme AChE in AD. Biochemical examinations of brain tissue samples obtained from AD animal models and human patients have revealed reduced ChAT activity and increased AChE activity in multiple brain areas (Bailey and Lahiri, 2012; Atukeren et al., 2017; Zheng et al., 2018). Supplementation with exogenous ChAT and/or AChE inhibitors (AChEIs) therefore represents a potential therapeutic strategy against AD.

The role of cholinergic receptors, i.e., nicotinic and muscarinic receptors in cholinergic neurotransmission, is not negligible. Muscarinic acetylcholine receptors (mAChRs) belong to the GPCR family and comprise the M1 and M2 subfamilies with a total of five subtypes. M1 receptors (M_1_, M_3_, and M_5_ mAChRs) are located in the postsynaptic membrane and couple to the Gα_q/11_ protein (Berizzi et al., 2018), whereas M2 receptors (M_2_ and M_4_ mAChRs) act as auto-receptors of the presynaptic membrane to inhibit ACh synthesis and release and couple to the Gα_i/o_ protein (Santiago and Abrol, 2019). M_1_ mAChR is highly expressed in the frontal cortex and hippocampus, and reduction of its levels exacerbates AD-like pathology and cognitive decline (Shiozaki et al., 2001; Tsang et al., 2006). These findings have more recently been confirmed in animal studies. Data from experiments in 3 × Tg-AD and Tg-SwDI mice indicated that ablating M_1_ mAChR promoted tau hyperphosphorylation and amyloidogenic processing, which were attributed to changes in PKC and GSK-3β activities, as well as increasing the astrocytic and microglial response associated with Aβ plaques (Medeiros et al., 2011). Consistent with this, the loss of M_1_ mAChR also resulted in increased levels of brain Aβ and greater accumulation of amyloid plaques in APP/PS1 transgenic mice (Davis et al., 2010). By contrast, activation of M_1_ mAChR was found to regulate Aβ neurotoxicity and tau pathology and reverse cognitive deficits through activation of PKC and inactivation of GSK-3β in a Gα_q/11_-dependent manner (Farias et al., 2004; Caccamo et al., 2006). Recently, based on knowledge of M_1_ mAChR, a variety of agonists (e.g., HTL9936, Brown et al., 2021) and positive allosteric modulators (e.g., VU0486846, Abd-Elrahman et al., 2021) have been designed and synthesized to alleviate the pathology of AD and improve cognitive function (Table 1).

**TABLE 1:** Muscarinic acetylcholine receptors reported to be involved in Alzheimer’s disease.

GPCRs	Subtype	Agent	Subject	Second messenger	Mode of action	References
M1 receptors	M_1_ or M_3_ mAChR	Carbachol (A)	Cell	↑ PKC	↑ sAPP ↓ Aβ	Nitsch et al., 1992
			Cell	↑ PKC	↑ sAPP ↓ Aβ	Buxbaum et al., 1992
			Cell	↑ PKC	↑ sAPP ↓ Aβ	Hung et al., 1993
			Cell	↑ PKC ↓ GSK-3β	↓ Tau	Forlenza et al., 2000
			Cell and rat	ND	↑ sAPP ↓ Aβ	Qiu et al., 2003
			Cell	↑ PKC	↑ ADAM17 and sAPPα	Cisse et al., 2011
	M_1_ mAChR	77-LH-28-1 (A)	Mouse	ND	↑ AMPAR and p-GluA1_Ser845_ ↑ PSD-95 and cognition	Zhao L. X. et al., 2019
		AF102B (A)	Mouse	ND	↑ Cognition	Fisher et al., 1991
			Cell	ND	↓ Tau	Sadot et al., 1996
			Human	ND	↓ Aβ	Nitsch et al., 2000
			Mouse	↑ PKC and ERK1/2	↑ ADAM17 ↓ Aβ	Welt et al., 2015
		AF150 (S) (A)	Mouse	ND	↓ Tau	Genis et al., 1999
		AF267B (A)	Cell	↑ PKC ↓ GSK-3β	↑ Wnt and β-catenin ↓ Aβ neurotoxicity	Farias et al., 2004
			Mouse	↑ PKC and ERK1/2 ↓ GSK-3β	↑ ADAM17 and cognition ↓ Aβ and tau	Caccamo et al., 2006
		AF710B (A)	Mouse	↓ GSK-3β	↓ BACE1, p25/CDK5, Aβ_40/42_, amyloid plaques, tau and neuroinflammation ↑ Cognition	Fisher A. et al., 2016
			Rat	ND	↓ Aβ_42_ and neuroinflammation ↑ synaptic plasticity and cognition	Hall H. et al., 2018
		EUK1001 (A)	Mouse	ND	↓ Tau ↑ Cognition	Wang D. et al., 2011
			Cell and mouse	ND	↓ Aβ_42_ ↑ sAPPα and cognition	Li Z. et al., 2017
		BQCA (PAM)	Mouse	ND	↑ sAPPα and cognition	Shirey et al., 2009
			Mouse	ND	↑ Cognition	Puri et al., 2015
		Talsaclidine (A)	Human	ND	↓ Aβ_42_	Hock et al., 2003
		TBPB (A)	Cell	ND	↑ sAPPα↓ Aβ_40_	Jones et al., 2008
		VU0364572 (A)	Mouse	ND	↓ oAβ, Aβ_40/42_ ↑ Cognition	Lebois et al., 2017
		VU0486846 (PAM)	Mouse	ND	↓ BACE1, oAβ, amyloid plaques, and neuronal loss ↑ ADAM10, anxiety-like behavior and cognition	Abd-Elrahman et al., 2021
M2 receptors		Methoctramine (AN)	Mouse	ND	↓ sAPPβ and Aβ	Cheng et al., 2010
			Mouse	↑ PKC ↓ GSK-3β	↑ ACh ↓ Tau	Zhang et al., 2014

*77-LH-28-1, 1-[3-(4-butylpiperidin-1-yl)propyl]-1,2,3,4-tetrahydroquinolin-2-one; A, agonist; Aβ, amyloid-β; ACh, acetylcholine; ADAM, a disintegrin and metalloprotease; AF102B, cevimeline; AF150(S), 1-methylpiperidine-4-spiro-(2′-methylthiazoline); AF267B, (2S)-2-Ethyl-8-methyl-1-thia-4,8-diazaspiro[4.5]decan-3-one; AF710B, 1-(2,8-Dimethyl-1-thia-3,8-diazaspiro[4.5]dec-3-yl)-3-(1H-indol-3-yl)propan-1-one; AMPAR,α-amino-3-hydroxy-5-methyl-4-isoxazole propionic acid receptors; AN, antagonist; APP,β-amyloid precursor protein; BACE1,β-secretase; BQCA, benzylquinolone carboxylic acid; CDK5, cyclin-dependent kinase 5; ERK, extracellular regulated protein kinases; EUK1001, 3−[3−(3−florophenyl−2−propyn−1−ylthio)−1,2,5−thiadiazol-4-yl]-1,2,5,6-tetrahydro−1− methylpyridine oxalate; GSK-3β, glycogen synthase kinase-3β; mAChR, muscarinic acetylcholine receptor; ND, not determined; oAβ, Aβ oligomer; PAM, positive allosteric modulator; PKC, protein kinase C; PSD-95, postsynaptic density protein-95; sAPP, soluble amyloid precursor protein; sAPPα, soluble amino-terminal ectodomain of APP; sAPPβ, solubleβ fragment of APP; TBPB, 1-(1′-2-methylbenzyl)-1,4′-bipiperidin-4-yl)-1H-benzo[d]imidazol-2(3H)-one; VU0364572, trifluoroacetate salt; VU0486846,(R)-4-(4-(1H-Pyrazol-1-yl)benzyl)-N-((1S,2S)-2-hydroxycyclohexyl)-3,4-dihydro-2H-benzo[b][1,4]oxazine-2-carboxamide.*

Similar to the results obtained for M_1_ mAChR, data from experimental *in vitro* studies indicated that M_3_ mAChR might be involved in coupling with PLC-mediated hydrolysis of phosphatidylinositol-4, 5-bisphosphate and PKC activation to stimulate the release of sAPP and reduce levels of Aβ (Buxbaum et al., 1992; Nitsch et al., 1992). This suggested that the activity of M_3_ mAChR was associated with amyloidogenic processing. Furthermore, M_3_ mAChR may be involved in improving learning and memory in a manner dependent on receptor phosphorylation/arrestin rather than via G protein signaling (Poulin et al., 2010). Abnormalities of M2 auto-receptors in AD brains have been mainly found in the cortex and hippocampus (Mulugeta et al., 2003; Bellucci et al., 2006). Notably, ACh levels can be increased by blockade of presynaptic M2 receptors that regulate ACh release; treatment with an M2 receptor antagonist, i.e., SCH-72788, can increase ACh contents (Lachowicz et al., 2001). The role of M2 receptors in AD has been partially established using a GRK5-deletion animal model. GRK5 is a serine/threonine kinase whose dysfunction selectively impairs desensitization of presynaptic M2 receptors, causes M2 receptors hyperactivity, and inhibits ACh release, resulting in cognitive impairment and AD-like pathology (Liu et al., 2009). In APP transgenic mice, inhibition of hyperactivity of presynaptic M2 receptors by antagonist methoctramine has been shown to be an effective therapy for eliminating the increase in Aβ and tau pathology induced by GRK5 deficiency and promoting ACh release (Cheng et al., 2010; Zhang et al., 2014; Table 1). Collectively, these findings provide evidence that activation of M1 receptors and inhibition of M2 receptors have potential benefits in ameliorating neuropathological features resembling those of AD.

### Physical Exercise, Cholinergic System, and Alzheimer’s Disease

Exercise increases cholinergic input in the cortex and hippocampus. In the cerebral cortex, significantly increased ACh contents were observed in rats following walking for as little as 5 min (Kurosawa et al., 1993). This suggests that ACh is released in response to exercise stimulation, even if the exercise volume is not large. Teglas et al. (2019) and Xu et al. (2019) found that chronic exercise programs, i.e., months of treadmill running [40 min/session, 3 sessions/week at 60% maximal oxygen intake (VO_2max_)] or voluntary wheel running (1 h/session, 5 sessions/week), attenuated age-related reduction of cholinergic fibers, reduced malformations of cholinergic forebrain innervation, and prevented the loss of cholinergic inputs in the hippocampus. Concomitant with these alterations, improvements in cognitive and motor behaviors was recorded. Similarly, Itou et al. (2011) reported that voluntary wheel running could reverse age-associated impairments of cholinergic innervation in the hippocampus of nestin promoter-GFP transgenic mice. In addition, Hall and Savage (2016) and Hall J. M. et al. (2018) observed re-emergence of the cholinergic/nestin neuronal phenotype (i.e., ChAT^+^/nestin^+^ neurons) within the medial septum/diagonal band (MS-DB) following exercise training; this improvement may in part have been mediated by nerve growth factor (NGF). These findings represent possible mechanisms by which the cholinergic system could promote cognitive function in response to exercise.

Several studies have reported regulation of the cholinergic system by exercise in AD animal models. In aged APP/PS1 transgenic mice, 4 weeks of continuous non-shock treadmill running (20–60 min/session, 5 sessions/week) improved learning and memory in association with an increased number of cholinergic neurons in the MS-DB (Ke et al., 2011). In THY-Tau22 mice, long-term voluntary wheel running for 9 months reversed pathological reduction of ChAT^+^ neurons in the MS-DB; this was accompanied by improvements in tau pathology and neuroinflammation within the hippocampus (Belarbi et al., 2011). Moreover, where comparisons were possible, the effect size for exercise was generally comparable with that of donepezil (Pisani et al., 2021). In Aβ_25–35_-induced rats, both chronic aerobic and resistance exercise have similar effects to those of AChEIs, reducing AChE activity and reversing recognition memory deficits (Farzi et al., 2019). However, conflicting results have been obtained regarding changes in AChE activity after exercise intervention; some investigators found that chronic exercise could increase AChE activity altered by Aβ neurotoxicity to maintain cholinergic system activity (Schimidt et al., 2019, 2021; Dare et al., 2020). Such contradictory results clearly warrant further study.

Several researchers have described the effects of combined interventions on the cholinergic system in AD. Donepezil hydrochloride combined with swimming exercise (7 sessions/week for 4 weeks with no weight bearing) improved learning and memory in Aβ_1–40_-induced rats, and the mechanism was related to increased ChAT activity and decreased AChE activity in the cortex and hippocampus (Jiangbo and Liyun, 2018). Probiotics can modulate the inflammatory process, counteract oxidative stress, and modify gut microbiota and are considered to be among the best preventive measures against cognitive decline in AD (Naomi et al., 2021). A study found that mono or combined progressive treadmill running (5 sessions/week for 8 weeks) and probiotic (e.g., Bifidobacterium bifidum and Lactobacillus plantarum) treatment significantly increased levels of ACh in the brains of Aβ_1–42_-induced rats and reversed spatial learning impairment (Shamsipour et al., 2021). Moreover, combined interventions could modulate the activity of M_1_ mAChR in the brain. Grape seed proanthocyanidin extract (GSPE) has been shown to have a strong antioxidant effect, can protect the central nervous system from oxidative stress damage, and may have a role in alleviating AD-related cognitive impairment (Sun et al., 2019; Gao et al., 2020). A study demonstrated that administration of GSPE and swimming training (2 h/session, 5 sessions/week for 14 weeks with 3% weight bearing) either individually or in combination led to improvements in learning and memory with reduced AChE activity in the medial prefrontal cortex and hippocampus of adult and middle-aged rats. Moreover, both mRNA and protein levels of M_1_ mAChR were increased in the cortex and hippocampus, and activation of the ERK/CREB/BDNF pathway was observed following swimming training with GSPE treatment (Abhijit et al., 2017). Although other mechanisms may be involved, these findings in individual models of AD indicate the importance of regulation of the cholinergic system (especially cholinergic neurons, ChAT, AChE, ACh, and M_1_ mAChR) by physical exercise.

## Noradrenergic System

### Noradrenergic Disturbances in Alzheimer’s Disease

The locus coeruleus (LC) is the norepinephrine (NE)-containing nucleus in the brainstem and innervates into widespread brain regions. It is composed of noradrenergic neurons that project to different brain regions and supplies NE to the cortex, hippocampus, striatum, amygdala, cerebellum, and basal forebrain, among others (Schwarz and Luo, 2015). The integrity of the LC-NE system is critical for attention, arousal, and specific aspects of learning and memory, and its activation across the lifespan is a crucial determinant of later-life cognitive reserve (Sara, 2009; Wilson et al., 2013; Mather and Harley, 2016). However, the LC-NE system is especially vulnerable to toxins and infection (Mather and Harley, 2016). During aging, decline of the LC-NE system is associated with reduced cognitive abilities relating to episodic memory and reduced cognitive reserve (Betts et al., 2019). Cumulative evidence suggests that aberrant tau accumulation in the LC and noradrenergic dysfunction are critical early events in the progression of AD (Mravec et al., 2016; Rorabaugh et al., 2017; Matchett et al., 2021). Aβ aggregation can also cause axonal degeneration in LC neurons (Sakakibara et al., 2021). Ablation of the LC-NE system, in turn, further exacerbates Aβ and tau pathology and the resulting cognitive deficits (Chalermpalanupap et al., 2018; Jacobs et al., 2021) and neuroinflammation (Jardanhazi-Kurutz et al., 2011; Cao et al., 2021), setting up a vicious cycle. Consequently, targeting the LC-NE system may have significant therapeutic potential in AD.

Notably, NE is widely regarded as a mediator of cognitive regulation in multiple neurodegenerative diseases, including AD (Holland et al., 2021). Animal studies suggest a progressive reduction of NE levels within the hippocampus, cortex, and cerebellum during the development of AD (Francis et al., 2012; Rorabaugh et al., 2017); these changes have been found to coincide with altered expression of BDNF and to precede the onset of cognitive and behavioral impairments (Francis et al., 2012). NE also has a central role in regulating Aβ production and Aβ-related pathologies. At the molecular level, it can interact with Aβ and inhibit Aβ generation (Liu et al., 2017; Zou et al., 2019). Furthermore, NE can reduce Aβ-induced neurotoxicity via activating tyrosine kinase receptor B (TrkB) (Liu et al., 2015), alleviate neuroinflammation by downregulating the expression of inducible nitric oxide synthase and interleukin 1β (IL-1β) (Heneka et al., 2002), and attenuate oxidative stress through limiting production of reactive oxygen species (ROS) (Jhang et al., 2014). By contrast, deficiency of NE increases the Aβ burden and activation of microglia and astroglia, and decreases expression and activity of the Aβ degrading-enzyme neprilysin (Kalinin et al., 2007). Thus, NE may improve a wide range of physiological and pathophysiological processes in AD.

To exert its neuroprotective effect, NE binds to adrenergic receptors (ARs), which can be classified into three main categories (i.e., α_1_, α_2_, and β) and all belong to the GPCR family. In general, the α_1_-AR subtypes (i.e., α_1A_, α_1B_, and α_1D_) couple to Gα_q/11_ (Chen and Minneman, 2005) and Gα_12/13_ (Maruyama et al., 2002) proteins, whereas the α_2_-AR subtypes (i.e., α_2A_, α_2B_, α_2C_, and α_2D_) couple to the Gα_i/o_ protein (Reynolds et al., 2005). Several studies have suggested that inhibition of α_1_-AR and/or α_2_-AR activity may represent a new strategy for anti-Aβ therapy. Previous studies showed that treatment with an α_1_-AR antagonist, i.e., prazosin or doxazosin, could reduce the generation of Aβ in N2a cells, alleviate neuroinflammation, and prevent memory deficits in APP23 transgenic mice (Katsouri et al., 2013), as well as protecting hippocampal slices from Aβ neurotoxicity through prevention of GSK-3β activation and tau hyperphosphorylation in an *in vitro* model of AD (Coelho et al., 2019). Similar to α_1_-AR, treatment with α_2_-AR antagonists including fluparoxan (Scullion et al., 2011), dexefaroxan (Francis et al., 2012), and mesedin (Melkonyan et al., 2017) was found to be beneficial for improving AD-like pathological mechanisms (Table 2). In addition, increased activity of α_2A_-AR was observed in AD patients and mouse models (Zhang F. et al., 2020). APP enhances the surface retention of α_2A_-AR and the intensity and duration of its signaling (Zhang et al., 2017); then, α_2A_-AR reduces Golgi localization of APP and concurrently promotes APP distribution in endosomes and cleavage by BACE1 (Chen et al., 2014). It follows that α_2A_-AR could serve as a key biological target for Aβ generation. Moreover, α_2A_-AR may represent a bridge between Aβ and tau pathology. One study demonstrated that oAβ bind to an allosteric site on α_2A_-AR to redirect NE-elicited signaling and thus to increase tau hyperphosphorylation, which depends on GSK-3β signaling (Zhang F. et al., 2020). The aforementioned results indicate that activation of α_2A_-AR may aggravate the pathological development of AD. It is necessary to further verify whether its genetic or pharmacological blockade could reduce Aβ production and Aβ-related neuropathology.

**TABLE 2:** Adrenergic receptors reported to be involved in Alzheimer’s disease.

GPCRs	Subtype	Agent	Subject	Second messenger	Mode of action	References
α_1_-AR		Doxazosin (AN)	Cell	↑ EGFR and Akt ↓ GSK-3β	↓ Aβ neurotoxicity and tau	Coelho et al., 2019
		Prazosin (AN)	Cell and mouse	ND	↓ Aβ_40_ and neuroinflammation ↑ sAPPα, neuronal number and cognition	Katsouri et al., 2013
α_2_-AR		Dexefaroxan (AN)	Mouse	ND	↑ BDNF and cognition →Aβ	Francis et al., 2012
		Fluparoxan (AN)	Mouse	ND	↑ Cognition →Amyloid plaque and neuroinflammation	Scullion et al., 2011
		Mesedin (AN)	Mouse	ND	↓ Aβ and neuroinflammation ↑ neurogenesis, neuronal maturation, neuronal and astroglial protection	Melkonyan et al., 2017
	α_2A_-AR	BRL-44408 (AN)	Mouse	ND	↓ Aβ↑ Cognition	Chen et al., 2014
β-AR		Carvedilol (AN)	Rat	ND	↓ Oxidative damage ↑ Cognition	Kumar et al., 2011
			Mouse	ND	↓ oAβ↑ Cognition	Wang J. et al., 2011
			Cell	ND	↓ Aβ neurotoxicity, oxidative stress and apoptosis	Liu and Wang, 2018
		Isoproterenol (A)	Rat	↑ PKA	↑ Tau and oxidative stress	Wang et al., 2004
			Rat	↑ PKA, CaMKII and CDK5 ↓ PP2A	↑ Tau ↓ Cognition	Sun et al., 2005
			Cell and mouse	ND	↑γ-secretase, PS1 and Aβ	Ni et al., 2006
			Cell	↑ ERK1/2 and p38 ↓ NF-κB	↓ Aβ_42_ ↑ IDE	Kong et al., 2010
			Cell and mouse	ND	↓ Tau	Soeda et al., 2015
			Mouse	ND	↓ Microglial inflammation	Xu et al., 2018
		Propranolol (AN)	Mouse	↑ Akt ↓ JNK and GSK-3β	↓ Aβ and tau ↑ IDE and cognition	Dobarro et al., 2013a
				↑ Akt and GSK-3β	↓ Aβ_42_, BACE1, and tau ↑ IDE, BDNF, SYP, and cognition	Dobarro et al., 2013b
	β_1_-AR	Xamoterol (A)	Mouse	↑ PKA and CREB	↑ Cognition	Coutellier et al., 2014
			Cell and mouse	↑ cAMP	↓ Aβ, tau, and neuroinflammation	Ardestani et al., 2017
	β_2_-AR	Clenbuterol (A)	Mouse	ND	↑ Aβ	Yu et al., 2010
			Mouse	ND	↓ Aβ and p-APP_Thr668_ ↑ Synaptic plasticity, neurogenesis, and cognition	Chai et al., 2016
			Cell and mouse	ND	↓ Aβ and p-APP_Thr668_ ↑α-secretase, synaptic plasticity and cognition	Chai et al., 2017
		Formoterol (A)	Mouse	↓ GSK-3β	↓ Oxidative stress, apoptosis and neuroinflammation ↑ Cognition	Abdel Rasheed et al., 2018
		ICI-118551 (AN)	Mouse	ND	↓ Aβ	Ni et al., 2006
			Mouse	ND	↓ Aβ	Yu et al., 2010
			Mouse	ND	↑ Aβ, Amyloid plaques and tau ↓ Cognition	Branca et al., 2014
			Mouse	ND	↑ Aβ and p-APP ↓α-secretase, synaptic plasticity and cognition	Wu et al., 2017
		Terbutaline (A)	Rat	↑ cAMP and PKA	↑ LTP	Wang et al., 2009
	β_3_-AR	CL-316243 (A)	Chick	ND	↑ Cognition	Gibbs et al., 2010
			Mouse	ND	↓ Insoluble Aβ_42_/Aβ_40_ ratio ↑ Cognition	Tournissac et al., 2021

*A, agonist; Aβ, amyloid-β; AN, antagonist; APP,β-amyloid precursor protein; Akt, protein kinase B; AR, adrenergic receptor; BACE1,β-secretase; BDNF, brain-derived neurotrophic factor; BRL-44408, (2-[2H-(1-methyl-1,3-dihydroisoindole) methyl]-4,5-dihydroimidazole); cAMP, cyclic adenosine monophosphate; CaMKII, Calcium/calmodulin-dependent protein kinase II; CDK5, cyclin-dependent kinase 5; CL-316243, 5-[(2R)-2-[[(2R)-2-(3-chlorophenyl)-2-hydroxyethyl]-amino]propyl]-1,3-benzodioxole-2,2-dicarboxylate; CREB, cAMP-response element binding protein; EGFR, epidermal growth factor receptor; ERK, extracellular regulated protein kinases; GSK-3β, glycogen synthase kinase-3β; ICI-118551, 3-(isopropylamino)-1-((7-methyl-2,3-dihydro-1H-inden-4-yl)oxy)butan-2-ol hydrochloride; IDE, insulin degrading enzyme; JNK, C-Jun kinase enzyme; LTP, long term potentiation; ND, not determined; NF-κB, nuclear factor kappa-B; oAβ, Aβ oligomer; p38, p38 mitogen-activated protein kinases; PKA, cyclic-AMP dependent protein kinase A; PP2A, protein phosphatase-2A; PS1, presenilin 1; SYP, synaptophysin.*

β-ARs are crucial targets for increasing synaptic plasticity and maintaining learning and memory (Goodman et al., 2021); however, the total number of β-ARs is significantly reduced in AD brains (Kalaria et al., 1989). NE may exert pleiotropic neuroprotective effects via its action on β-AR signaling in various cell types. Activation of β-AR signaling may be responsible for the activation of CREB and the induction of NGF and BDNF to protect against Aβ neurotoxicity in neurons (Counts and Mufson, 2010). In microglia-like cells, NE suppresses Aβ-induced toxicity and production of monocytic chemotactic protein-1, a pro-inflammatory chemokine, through activating β-AR signaling, accompanied by activation of the cAMP/PKA/CREB pathway (Yang et al., 2012). Furthermore, as shown in Table 2, accumulating evidence from pharmacological intervention studies confirms that β-ARs mediate distinct functions related to different aspects of AD pathology. Some previous studies, for instance, have reported that application of isoproterenol, a non-selective β-AR agonist, worsened Aβ and tau pathology; however, other studies have demonstrated the opposite effect. Hence, the molecular mechanism of the differential effect of the same β-AR agonist in different models of AD needs to be studied. The regulatory roles of different β-AR subtypes in AD pathology should also be considered.

There are three β-AR subtypes (β_1_, β_2_, and β_3_) in the brain that couple to the Gα_s_ protein (Liu et al., 2019; Lee et al., 2020; Su et al., 2020). Recently, the contribution of the β_1_- and β_3_-ARs to AD pathology and cognitive function has been a subject of interest. Animal studies have revealed that selective activation of β_1_-AR activates the cAMP/PKA/CREB pathway to rescue social memory deficit in APP mice (Coutellier et al., 2014), as well as inhibiting the expression of neuroinflammatory markers (e.g., ionized calcium binding adapter molecule 1) and reducing Aβ and tau pathology in 5 × FAD mice (Ardestani et al., 2017). Moreover, treatment with CL-316243, a β_3_-AR agonist, could reduce Aβ pathology and reverse memory loss (Gibbs et al., 2010; Tournissac et al., 2021; Table 2). Therefore, the current consensus is that pharmacological activation of β_1_- and β_3_-ARs is beneficial in AD. However, prior research on β_2_-AR in AD resulted in seemingly contradictory findings. Some studies showed that activation of β_2_-AR accelerated amyloid plaque formation, and that this beneficial effect could be reversed by antagonist ICI-118551 (Ni et al., 2006; Yu et al., 2010). Conversely, other studies have demonstrated that treatment with highly selective β_2_-AR agonists such as clenbuterol can reduce Aβ levels, promote hippocampal neurogenesis, enhance synaptic plasticity, and improve neuronal death and microglial inflammation (Chai et al., 2016, 2017), whereas application of ICI-118551 exacerbates Aβ and tau neuropathology and cognitive deficits (Branca et al., 2014; Wu et al., 2017). Further studies are warranted to confirm and explain these contradictory observations.

### Physical Exercise, Noradrenergic System, and Alzheimer’s Disease

Previous studies in animals have shown that central noradrenergic neurons are activated in response to exercise training and participate in thermoregulation during exercise (Ohiwa et al., 2006; Rodovalho et al., 2020). Acute running increases noradrenergic activity, and the longer the running time, the longer the duration of central activation in the recovery period (Pagliari and Peyrin, 1995a,b). In the course of chronic exercise intervention, rodents with long-term exercise training experience showed significant increases (approximately 26%) in levels of NE in the brain (Ostman and Nyback, 1976). Consistent with this, progressive treadmill running for 8 weeks in rodents was accompanied by brain noradrenergic adaptations and increases in NE levels in the areas of NE cell bodies and the spinal cord (Dunn et al., 1996). Based on this information, a single bout of exercise appears to temporarily increase central noradrenergic activity, and the cumulative effect of long-term regular exercise leads to a significant increase in the levels of NE.

Salivary α-amylase (sAA), a non-invasive biomarker of central noradrenergic activity, is a promising avenue for characterizing the arousal-mediated effects of exercise on cognition (Weiss et al., 2019). A study demonstrated that 6 min of stationary bicycle exercise at 70% VO_2max_ significantly enhanced memory consolidation in both patients with amnestic MCI and cognitively normal individuals through activating the noradrenergic system (as determined by measuring sAA) (Segal et al., 2012). In another study, patients with MCI who underwent a chronic mind-body exercise program [60 min/session, 3 sessions/week for 24 weeks at 55–75% heart rate reserve (HRR)] showed a significant increase in intrinsic functional connectivity in the LC-NE system and improvements in cognitive performance, as measured by magnetic resonance imaging (MRI) scans and the Montreal Cognitive Assessment (Liu et al., 2021). These findings suggest that activation of the noradrenergic system by exercise improves cognitive performance of individuals in the prodromal stage of AD.

Studies in animal models found that exercise could also effectively reduce the activity of α_2_-AR and increase the activity of β-ARs. Spontaneously-Running-Tokushima-Shikoku rats, an animal model for high levels of wheel-running activity, showed decreased hippocampal monoamine oxidase A levels and increased extracellular NE levels, and the elevation of NE levels caused downregulation of α_2_-AR (Morishima et al., 2006). Furthermore, the affinity of this receptor in the nucleus tractus solitarius was reduced in trained rats compared with sedentary animals (De Souza et al., 2001). These results indicate that long-term exercise may lead to reduced affinity of α_2_-AR in multiple brain regions. Considering the anti-AD effect produced by inhibiting α_2_-AR, the potential of α_2_-AR-mediated exercise to improve AD deserves future exploration. In addition to the findings for α_2_-AR, Ebrahimi et al. (2010) confirmed that exercise enhanced learning and memory through β-AR-dependent pathways by administering propranolol to mice. Indeed, previous studies have proposed that an intact noradrenergic system, especially activation of β-ARs by NE, serves as a vital link in the upregulation of BDNF expression by exercise (Garcia et al., 2003; Ma, 2008). BDNF can induce the expression of thioredoxin-1 (TRX-1) via the TrkB/Akt/CREB pathway (Bai et al., 2019). TRX-1 is a disulfide-reducin-system low-molecular-weight protein with redox properties, levels of which are significantly reduced in AD brains (Akterin et al., 2006). Increased TRX-1 levels can alleviate endoplasmic reticulum stress, oxidative stress, and apoptosis in AD (Guo et al., 2021). Recently, an experimental study showed that a treadmill running program (60 min/session, 6 sessions/week for 3 weeks) increased the content of TRX-1 in the hippocampus of mice and activated the ERK1/2/β-catenin/T-cell factor pathway, which in turn promoted hippocampal cell proliferation and neurogenesis (Kim and Leem, 2019). Notably, Kim and Leem (2019) proposed a hypothesis regarding the signaling that links exercise, β_2_-AR, BDNF, and TRX-1; that is, exercise may promote the expression and interaction of BDNF and TRX-1 through activating the β_2_-AR/cAMP/PKA pathway. A study further to test this hypothesis subjected an animal model of cognitive impairment induced by a high-fat diet to a treadmill running program (30 min/session, 5 sessions/week for 23 weeks at 40–50% VO_2peak_); activation of the β_2_-AR/cAMP/PKA pathway, increased expression of TRX-1 and BDNF, inhibition of microglial activation, decreased expression of inflammatory markers, and reduction of oxidative stress markers in the dentate gyrus of the hippocampus were observed (Han et al., 2019). These results suggest that exercise alleviates neuroinflammation and oxidative stress potentially through a signaling cascade involving β_2_-AR, BDNF, and TRX-1. However, the relationship among the these three factors needs to be validated in AD animal models and patients.

## Serotonergic System

### Serotonergic Disturbances in Alzheimer’s Disease

Serotonergic neurotransmission is dependent on the synthesis and release of the neurotransmitter serotonin [5-hydroxytryptamine (5-HT)], and the serotonergic projections from the dorsal raphe nucleus (DRN) have widespread ramifications throughout the brain, including the frontal cortex, temporal cortex, and hippocampus (Vertes, 1991; Vertes et al., 1999). Aging exerts complex effects on the central serotoninergic system. Impaired serotonergic neurotransmission and altered expression of 5-HT transporter (5-HTT) and 5-HT receptors (5-HTRs) have been observed in multiple brain regions, although the number of serotonergic neurons did not change significantly (Rodriguez et al., 2012). Several studies have demonstrated that dysfunction of the serotonergic system is linked to the development of AD pathology (Rodriguez et al., 2012; Tajeddinn et al., 2016b; Jankowska et al., 2018). The number of serotonergic neurons in the DRN (Aletrino et al., 1992; Lyness et al., 2003) and the contents of 5-HT, 5-HTT, and its metabolite 5-hydroxyindoleacetic acid (5-HIAA) in the cortex and hippocampus have been shown to be significantly reduced in AD brains (Palmer et al., 1987; Thomas et al., 2006; Vermeiren et al., 2014). Furthermore, lower concentrations of 5-HT in cerebrospinal fluid (Lanctot et al., 2002) and platelets (Prokselj et al., 2014; Tajeddinn et al., 2016a) have been observed in patients with AD compared with controls. Treatment studies aim to increase serotonergic tone; selective serotonin reuptake inhibitors including escitalopram (Ehrhardt et al., 2019), citalopram (Porsteinsson et al., 2014), and fluoxetine (Xie et al., 2019) have been found to have beneficial effects on psychiatric symptoms and cognitive impairment in patients with AD.

Increasing attention has been paid to the functions of 5-HTRs and their impact on the pathophysiology of AD. In general, 5-HTRs constitutes seven subfamilies, which can be split into a total of 14 subtypes (i.e., 5-HT_1A–1F_R, 5-HT_2A–2c_R, 5-HT_3_R, 5-HT_4_R, 5-HT_5A–5B_R, 5-HT_6_R, and 5-HT_7_R). Except for 5-HT_3_R, these receptors belong to the GPCR family (Sharp and Barnes, 2020). The 5-HT_1_R and 5-HT_5_R subfamilies couple to the Gα_i/o_ protein and mainly inhibit PKA signaling (Francken et al., 2000; Rojas et al., 2017). 5-HT_1_R members are expressed in large quantities in the hippocampus and have a significant role in the regulation of memory processes (Ogren et al., 2008). 5-HT_1A_R is a well-studied member of this subfamily and shows overexpression under Aβ stimulation (Verdurand et al., 2011, 2016). Treatment with 5-HT_1A_R antagonists (e.g., NAD-299 and WAY-100635) has been shown to reduce amyloid plaque deposition, increase levels of hippocampal BDNF, alleviate neuroinflammation and oxidative stress, and improve cognitive deficits in individual animal models of AD (Afshar et al., 2018, 2019; Wang et al., 2020; Table 3). These results imply that 5-HT_1A_R, in response to specific ligands, is involved in the regulation of AD pathology through multiple pathways.

**TABLE 3:** Serotonergic receptors reported to be involved in Alzheimer’s disease.

GPCRs	Subtype	Agent	Subject	Second messenger	Mode of action	References
5-HT_1_R	5-HT_1A_R	8-OH-DPAT (A)	Cell	↑ PI3K and Akt ↓ GSK-3β	↓ Tau	Wang et al., 2016
		NAD-299 (AN)	Rat	ND	↓ Amyloid plaques and neuronal loss ↑ BDNF and cognition	Afshar et al., 2018
			Rat	ND	↓ Oxidative stress and neuronal loss	Afshar et al., 2019
			Rat	ND	↓ Neuronal apoptosis	Shahidi et al., 2019a
		WAY-100635 (AN)	Mouse	↓ NF-κB	↓ Neuroinflammation ↑ Cognition and neuronal survival	Wang et al., 2020
	5-HT_1B_R	EG (A)	Cell	↑ ERK1/2	↓ Neuroinflammation and neural death	Yang et al., 2021
5-HT_2_R	5-HT_2A_R	Desloratadine (AN)	Mouse	↑ cAMP, PKA, CREB, and Sirt1	↓ Amyloid plaques and neuroinflammation	Lu et al., 2021
		Pimavanserin or M100907 (IA)	Mouse	↑ ERK	↓ Aβ↑α-secretase and cognition	Yuede et al., 2021
		TCB-2 (A)	Rat	ND	↓ Amyloid plaques and neuronal loss ↑ BDNF and cognition	Afshar et al., 2018
			Rat	ND	↓ Oxidative stress and neuronal loss	Afshar et al., 2019
			Rat	ND	↓ Neuronal apoptosis	Shahidi et al., 2019a
	5-HT_2C_R	Dexnorfenfluramine (A)	Guinea pig	ND	↑ sAPP ↓ Aβ	Arjona et al., 2002
		RO-60-0175 (A)	Cell	ND	↑ NEP	Tian et al., 2015
5-HT_4_R		BIMU8 (A)	Rat	ND	↓ Neuronal apoptosis ↑ Synaptic plasticity and cognition	Hashemi-Firouzi et al., 2021
		ML10302 (A)	Mouse	ND	↑ sAPPα	Cachard-Chastel et al., 2007
			Mouse	ND	↑ sAPPα	Russo et al., 2009
		Prucalopride (A)	Cell	↑ PKA	↑ sAPPα	Robert et al., 2001
			Mouse	ND	↑ sAPPα	Cachard-Chastel et al., 2007
		RS-67333 (A)	Cell and mouse	ND	↑ sAPPα and neuron survival ↓ Aβ	Cho and Hu, 2007
			Cell and mouse	ND	↑ sAPPα, CTFα, and MMP-9 ↓ Aβ_40_ and amyloid plaques	Hashimoto et al., 2012
			Mouse	ND	↑ sAPPα and cognition ↓ Aβ_40/42_, amyloid plaques and neuroinflammation	Giannoni et al., 2013
			Mouse	ND	↓ Amyloid plaques and neuroinflammation ↑ Cognition	Baranger et al., 2017
		SSP-002392 (A)	Cell and mouse	↑ cAMP	↓ Aβ, BACE1, ADAM17, nicastrin, and neuroinflammation	Tesseur et al., 2013
5-HT_6_R		SB-258585 (AN)	Cell and mouse	↑β-arrestin2 and CDK5	↓ Aβ	Li X. et al., 2017
			Rat	ND	↓ Neuronal apoptosis ↑ Cognition	Hashemi-Firouzi et al., 2018
			Rat	ND	↑ Neuronal plasticity and cognition	Shahidi et al., 2019b
		EMD-386088 (A) or SB-399885 (AN)	Cell	ND	↓ Aβ neurotoxicity, oxidative stress and apoptosis ↑ Neurite outgrowth	Bokare et al., 2017
		SB-271046 (AN)	Mouse	ND	↓γ-secretase, Aβ and neuroinflammation ↑ Cognition	Yun et al., 2015
			Cell and mouse	ND	↑ Neuronal cilia morphology and cognition	Hu et al., 2017
5-HT_7_R		AS-19 (A)	Rat	ND	↑ Synaptic function ↓ Neuronal apoptosis	Hashemi-Firouzi et al., 2017
			Rat	ND	↓ Amyloid plaques and neuronal apoptosis ↑ Synaptic function and cognition	Shahidi et al., 2018

*8-OH-DPAT, 8-hydroxy-2-(di-n-propylamino)tetralin hydrobromide; A, agonist; Aβ, amyloid-β; ADAM, a disintegrin and metalloprotease; AN, antagonist; Akt, protein kinase B; APP,β-amyloid precursor protein; AS-19, (2S)-(+)-5-(1,3,5-TriMethylpyrazol-4-yl)-2-(diMethylaMino)tetralin; BDNF, brain-derived neurotrophic factor; BACE1,β-secretase; BIMU8, (endo-N-8-methyl-8-azabicyclo[3.2.1]oct-3-yl)-2,3-dehydro-2-oxo-3-(prop-2-yl)-1H-benzimid-azole-1-carboxamide; cAMP, cyclic adenosine monophosphate; CDK5, cyclin-dependent kinase 5; CREB, cAMP-response element binding protein; CTFα, C-terminal fragmentα; EG, emodin-8-O-β-d-glucopyranoside; EMD-386088, 5-chloro-2-methyl-3-(1,2,3,6-tetrahydro-4-pyridinyl)-1H-indole hydrochloride; ERK, extracellular regulated protein kinases; GSK-3β, glycogen synthase kinase-3β; M100907, (R)-(+)-(2,3-dimethoxyphenyl)-1-[2-(4-fluorophenyl)ethyl]-4-piperidine methanol; IA, inverse agonist; ML10302, 2-piperidinoethyl 4-amino-5-chloro-2-methoxybenzoate; MMP-9, matrix metalloprotein 9; NAD-299, (R)-3-N,N-dicyclobutylamino-8 fluoro-3,4-dihydro-3H-1-benzopyran-5-carboxamide hydrogen (2R,3R)-tartrate monohydrate; ND, not determined; NEP, neprilysin; NF-κB, nuclear factor kappa-B; PKA, cyclic-AMP dependent protein kinase A; PI3K, phosphatidylinositol 3-kinase; RO-60-0175, (S)-2-(chloro-5-fluoro-indol-l-yl)-1-methylethylamine fumarate; RS-67333, 1-(4-Amino-5-chloro-2-methoxyphenyl)-3-[1-butyl-4-piperidinyl]-1-propanone hydrochloride; sAPPα, soluble amino-terminal ectodomain of APP; SB-258585, 4-Iodo-N-[4-methoxy-3-(4-methyl-piperazin-1-yl)-phenyl]-benzen esulphonamide; SB-271046, 5-Chloro-N-(4-methoxy-3-piperazin-1-yl-phenyl)-3-methyl-2-benzothiophenesulfon-amide; SB-399885, N-[3,5-dichloro-2-(methoxy)phenyl]-4-(methoxy)-3-(1-piperazinyl)benzenesulfonamide; SIRT 1, silent mating type information regulation 2 homolog-1; SSP-002392, (4-amino-5-chloro-2,3-dihydro-benzofuran-7-carboxylic acid [3-hydroxy-1-(3-methoxy-propyl)-piperidin-4ylmethyl]-amide); TCB-2, (7R)-3-bromo-2, 5-dimethoxy-bicyclo[4.2.0]octa-1,3,5-trien-7-yl]methanamine; WAY-100635, [O-methyl-3H]-N-(2-(4-(2-methoxyphenyl)-1-piperazinyl)ethyl)-N-(2- pyridinyl)cyclohexanecarboxamide trihydrochloride.*

Unlike the above two subfamilies, members of the 5-HT_2_R subfamily couple to the Gα_q/11_ protein (Odagaki et al., 2017) and are strongly associated with Aβ pathology, especially 5-HT_2A_R and 5-HT_2C_R. An imaging study has revealed prominent reductions in neocortical 5-HT_2_R in patients with AD (Blin et al., 1993). The binding of 5-HT_2A_R is significantly decreased in the brains of AD animal models (Holm et al., 2010) and human patients (Lai et al., 2005; Marner et al., 2012). Molecular biochemistry studies have disclosed that 5-HT could induce the release of sAPP through activation of 5-HT_2A_R and 5-HT_2C_R (Nitsch et al., 1996). Although there is evidence to suggest that the 5-HT_2A_R and 5-HT_2C_R modulate sAPP secretion *in vitro* and *in vivo* (Table 3), further studies are required to determine whether this effect is mediated by a change in α- or β-secretase activity and whether this effect correlates with a change in Aβ generation. Furthermore, studies have found that administration of 5-HT_2A_R-selective ligands stimulated the autophagy process of microglia, enhanced the phagocytosis of Aβ (Lu et al., 2021), and reduced amyloid plaques (Afshar et al., 2018). Hence, 5-HT_2A_R may also be a key target for regulating Aβ clearance and degradation.

The remaining subfamilies (5-HT_4_R, 5-HT_6_R, and 5-HT_7_R) couple to the Gα_s_ protein and activate PKA signaling (Fisher J. R. et al., 2016). 5-HT_4_R and 5-HT_6_R are mainly located in brain regions involved in cognitive processes (King et al., 2008). Neurochemical and behavioral studies have demonstrated that activation of 5-HT_4_R or blockade of 5-HT_6_R improved cognitive performance (Quiedeville et al., 2015). Recently, 5-HT_4_R agonists and 5-HT_6_R antagonists have attracted interest with respect to AD treatment and have been widely investigated from a drug discovery perspective. Studies *in vitro* and *in vivo* suggest that activation of 5-HT_4_R by agonists (e.g., ML10302, prucalopride, and RS-67333) increases levels of neuroprotective sAPPα, reduces amyloid plaque deposition, and rescues cognitive deficits through the classical cAMP/PKA pathway and non-classical pathway. In addition, inhibition of 5-HT_6_R by antagonists (e.g., SB-258585, SB-399885, and SB-271046) can inhibit Aβ generation, and protect neurons from Aβ-induced neurotoxicity, neuroinflammation, oxidative stress, and apoptosis (Table 3). Preclinical and early clinical studies of 5-HT_4_R agonism and 5-HT_6_R antagonism are being conducted to investigate the ability of these approaches to alleviate cognitive deficits associated with AD (Ivachtchenko et al., 2016; Nirogi et al., 2021). 5-HT_7_R, the most recently discovered receptor of 5-HT, has been shown to regulate individual cognition (Gasbarri and Pompili, 2014). Chronic treatment with AS-19, a selective 5-HT_7_R agonist, could prevent cognitive deficits by alleviating Aβ plaque accumulation and neuronal apoptosis and improving neuronal plasticity (Hashemi-Firouzi et al., 2017; Shahidi et al., 2018). Furthermore, a recent study revealed that 5-HT_7_R is an important target for the treatment of tauopathy (Labus et al., 2021). Collectively, 5-HT_4_R, 5-HT_6_R, and 5-HT_7_R may represent novel therapeutic targets for the treatment and prevention of AD.

### Physical Exercise, Serotonergic System, and Alzheimer’s Disease

The positive effects of physical exercise on emotional and cognitive performance through activation of the serotonergic system can be summarized as follows. First, exercise enhances the function of the serotonergic system via increases in numbers and activity of serotonergic neurons in the DRN. Treadmill running exercise, whether performed acutely for 30 min or chronically for 3 or 8 weeks, increased the number and activity of serotonergic neurons in the DRN of rodent models (Hong et al., 2015; Otsuka et al., 2016; Ge and Dai, 2020). In particular, 4 weeks of progressive treadmill running (20–60 min/session, 5 sessions/week) selectively improved spatial learning and memory in association with an increase in numbers of serotonergic neurons in the DRN of aged APP/PS1 transgenic mice (Ke et al., 2011); this exercise paradigm also reduced Aβ levels and abnormal microglia activation, but not enough to reduce the plaque loading in the hippocampus.

Second, exercise increases the availability of 5-HT precursor tryptophan (TRP) and TRP hydroxylase (TPH), and increases levels of 5-HT and 5-HTAA. Studies in humans suggest that a bout of exercise, i.e., 35 min of graded exercise or 60 min of treadmill exercise, can increase serum TRP and 5-HT levels and exert pro-cognitive and antidepressant effects (Melancon et al., 2012; Zimmer et al., 2016). Additional studies found that 5-HT released during yoga and meditative practices activated an alternate cleavage of APP to produce a fragment with known neurotrophic effects, giving it the unique ability to inhibit the oAβ production cycle in an *in vitro* AD model (Hassan et al., 2018). Data from animal studies indicate that exercise significantly increases the synthesis and metabolism of central 5-HT (Dey et al., 1992), and is required for the exercise-induced neurogenic response, especially adult hippocampal neurogenesis (Klempin et al., 2013, 2018). Furthermore, saffron combined with endurance exercise increased levels of hippocampal 5-HIAA, and this change was associated with improved short-term memory (Akbari-Fakhrabadi et al., 2021). Regulation of central 5-HT synthesis and metabolism by exercise may represent a therapeutic opportunity in depression and age-related cognitive decline. Isolation stress accelerates the onset of AD (Huang et al., 2015; Peterman et al., 2020), reducing TPH and 5-HT expression in the DRN and promoting apoptosis in the hippocampus, leading to anxiety and memory decline during old age, whereas a swimming exercise program (30 min/session, 5 sessions/week for 4 weeks) reversed these changes (Park et al., 2020). Moreover, three different forms of exercise (i.e., treadmill exercise, involuntary exercise and voluntary exercise, 5 sessions/week for 4 weeks) could all improve cognitive and behavioral functions by increasing levels of hippocampal 5-HT in a vascular dementia rat model (Zhang L. et al., 2020).

Third, exercise can regulate the expression of the serotonergic receptor and the activity of its downstream signaling pathway to improve impaired cognitive ability and abnormal emotion. Notably, as the hippocampal neurons mainly express type II AC, which is not regulated by the Gα_i/o_ subunit but is activated by the Gβγ subunit, the cAMP/PKA pathway is activated by 5-HT_1A_R (Albert and Vahid-Ansari, 2019). Exercise improves cognitive function by increasing the expression of 5-HT_1A_R in hippocampal neurons, which is potentially associated with BDNF-related signaling. Results from several animal studies are consistent with this notion. For instance, Kim et al. (2015) reported that 4 weeks of treadmill running (30 min/session, 7 sessions/week) enhanced CREB phosphorylation and increased the expression of BDNF and TrkB via activation of 5-HT_1A_R in rat hippocampus neurons. Consistent with this, the improvements in learning and memory after rats underwent a chronic and progressive treadmill running program (30–60 min/session, 3 sessions/week for 14 weeks at 60–70% VO_2max_) were found to be due to increased expression of 5-HT, 5-HTT, 5-HT_1A_R, and BDNF in the hippocampal CA1 area (Pietrelli et al., 2018). In addition, treadmill exercise training (45 min/session, 3 sessions/week for 32 weeks) could ameliorate anxious/depressive-like behavior and attenuate fear-avoidance behavior deficits in TgF344-AD rats in the early stage of Alzheimer’s pathogenesis by increasing the expression of 5-HT and 5-HT_6_R in the cortex and hippocampus (Wu et al., 2020). Existing studies on the regulation of individual cognition by exercise through the regulation of serotonergic receptor function are still lacking, and more detailed evidence is necessary.

## Dopaminergic System

### Dopaminergic Disturbances in Alzheimer’s Disease

Dopamine (DA) is a major catecholamine neurotransmitter that projects dopaminergic signaling to the prefrontal cortex, hippocampus, striatum, nucleus accumbens, amygdala, and other areas from the ventral tegmental nucleus area (VTA) of the midbrain; it is mainly involved in emotion, behavior, and cognition, and in regulation of synaptic plasticity (Jay, 2003; Bjorklund and Dunnett, 2007). Aging is associated with a loss of dopaminergic function, which may originate from defects on multiple components, including loss of dopamine-producing neurons, atrophy of projection brain regions, and reduced density of dopamine receptors. These alterations result in the efficiency of dopaminergic projecting systems declines slowly during physiological aging (Ciampa et al., 2021; Gasiorowska et al., 2021). Cumulative evidence suggests that impaired dopaminergic neurotransmission is also involved in the pathological development of a variety of neurological disorders, including AD (Martorana and Koch, 2014; D’Amelio et al., 2018). In particular, loss of dopaminergic neurons in the VTA and/or substantia nigra pars compacta (SNpc) (Mann et al., 1987; Nobili et al., 2017) and significantly decreased levels and availability of DA in the cortex and hippocampus have been observed in AD animal models and human patients, where they led to severe synaptic dysfunction and cognitive deficits (Reinikainen et al., 1988; Trabace et al., 2007). In a recent animal study, Aβ decreased cortical DA levels and caused profound impairment of both long-term potentiation (LTP) and long-term depression, as well as recognition memory (Moreno-Castilla et al., 2016). In the Tg2576 mouse model of AD, dopaminergic neuron loss was shown to begin before Aβ plaque formation, resulting in reduced hippocampal DA outflow, which decreased neuronal synaptic plasticity and excitability and contributed to memory and reward dysfunction; nevertheless, these defects could be partially reversed with DA precursor levodopa (L-DOPA) supplementation (Nobili et al., 2017; Cordella et al., 2018). Thus, increasing DA levels moderately may ameliorate synaptic dysfunction and cognitive decline in AD.

In addition to improvements in neuroplasticity, the effects of DA on AD neuropathology include disruption of Aβ protofibril and inhibition of Aβ aggregation, as well as partial alleviation of neuroinflammation, and oxidative stress. On the one hand, DA can inhibit Aβ aggregation and disrupt Aβ fibrils in a dose-dependent manner (Huong et al., 2010; Liu et al., 2016). Recently, Chen et al. (2021) reported that DA disrupted Aβ protofibrils and prevented Aβ dimerization at the molecular level mostly through π-π stacking interactions with residues F4, H6, and H13; hydrogen-bonding interactions with negatively charged residues D7, E11, E22, and D23; and cation-π interactions with residue R5. This may be an important mechanism by which DA interferes with Aβ generation. On the other hand, DA and its derivatives significantly diminish neuroinflammation and oxidative stress triggered by lipopolysaccharides (LPS) and Aβ through decreasing levels of inflammatory mediators and upregulating expression of heme oxygenase-1, the enzyme responsible for production of antioxidants (Nam et al., 2018). Further research should determine the optimal dose-effect relationship for DA regulation of AD-like pathology.

Dopaminergic receptors, which are widely distributed in various brain regions, belong to the GPCR family and can be divided into D1-like receptors (i.e., DA_1_R and DA_5_R) and D2-like receptors (i.e., DA_2_R, DA_3_R, and DA_4_R) according to their biological and pharmacological properties (Beaulieu and Gainetdinov, 2011). D1-like receptors are widely distributed in the brain, couple to Gα_s_ and Gα_q/11_ proteins (Himmelreich et al., 2017), and regulate PKA and PLC signaling, whereas D2-like receptors couple to the Gα_i/o_ protein (Conley and Watts, 2013). Interestingly, a recent systematic review and network meta-analysis indicated that DA_1_R and DA_2_R levels were decreased in patients with AD compared with controls; the dopaminergic receptors were ranked as follows according to their correlation with AD from highest to lowest: DA_2_R, DA_3_R, DA_4_R, DA_5_R, and DA_1_R (Pan et al., 2019). As shown in Table 4, application of D1-like receptor agonists (e.g., L-stepholidine, L-theanine, and SKF-38393) reduced Aβ and tau pathology and significantly improved synaptic dysfunction and cognition. These results are largely consistent with the activation of PKA signaling. Furthermore, *in vitro* and *in vivo* experiments confirmed that application of selective DA_1_R agonist A-68930 significantly ameliorated neuroinflammation and mitochondrial dysfunction through adenine monophosphate activated protein kinase (AMPK)-related signaling pathways (Cheng et al., 2020, 2021). Together, these results indicate that activation of D1-like receptors represents an important strategy for prevention and treatment of AD-like pathology.

**TABLE 4:** Dopaminergic receptors reported to be involved in Alzheimer’s disease.

GPCRs	Subtype	Agent	Subject	Second messenger	Mode of action	References
D1-like receptor		L-stepholidine (A)	Cell and mouse	↑ PKA	↑ AMPAR and p-GluA1_Ser845_, synaptic function, and cognition	Hao et al., 2015
		L-theanine (A)	Mouse	↑ PKA	↑ Synaptic function and cognition	Zhu et al., 2018
		SKF-38393 (A)	Cell and rat	↑ PKA and CDK5 ↓ GSK-3β	↑ Tau	Lebel et al., 2009
			Mouse	↑ SFK	↓ oAβ neurotoxicity ↑ Synaptic function and cognition	Yuan Xiang et al., 2016
			Mouse	↑ p-CREB	↓ Aβ, BACE1 and neuronal apoptosis ↑ BDNF and cognition	Zang et al., 2018
		SKF81297 (A)	Rat	ND	↑ AMPAR and p-GluA1_Ser845,_ NMDAR and synaptic function	Jurgensen et al., 2011
	DA_1_R	A-68930 (A)	Cell and mouse	↑ AMPK	↓ Neuroinflammation and neuronal damage ↑ Cognition	Cheng et al., 2020
			Cell and mouse	↑ AMPK and PGC-1α	↓ Aβ, BACE1, tau, and mitochondrial dysfunction ↑ Cognition	Cheng et al., 2021
	DA_5_R	027075 (A)	Mouse	↑ cAMP	↓ Aβ, BACE1, PS1, and apoptosis ↑ NEP, cell differentiation, neurite length, and cognition	Shen et al., 2016
D2-like receptor	DA_2/3_R	Rotigotine (A)	Human	ND	↑ Cortical excitability and cholinergic transmission	Martorana et al., 2013
	DA_2_R	Levodopa or piribedil (A)	Cell and mouse	↑β-arrestin2	↑ Aβ and γ-secretase	Lu et al., 2017
		Quinpirole (A)	Cell and mouse	ND	↓ Aβ_42_ neurotoxicity	Melief et al., 2015

*A, agonists; A-68930, (1R, 3S)-1-aminomethyl-5,6-dihydroxy-3-phenylisochroman HCI; Aβ, amyloid-β; AMPAR,α-amino-3-hydroxy-5-methyl-4-isoxazole propionic acid receptors; AMPK, adenine monophosphate activated protein kinase; AN, antagonists; BACE1,β-secretase; cAMP, cyclic adenosine monophosphate; CDK5, cyclin-dependent kinase 5; CREB, cAMP-response element binding protein; GSK-3β, glycogen synthase kinase-3β; LTP, long term potentiation; ND, not determined; NEP, neprilysin; NMDA, N-methyl-D-aspartate receptors; oAβ, Aβ oligomer; PGC-1α, peroxisome-proliferator-activated receptorγcoactjvator-1α; PKA, cyclic-AMP dependent protein kinase A; PS1, presenilin 1; SFK, Src-family tyrosine kinases; SKF-38393, (7,8-dihydroxy-1-phenyl-2,3,4,5-tetrahydro-1 H-3-benzazepine); SKF81297, (6-chloro-7,8-dihydroxy-1-phenyl-2,3,4,5- tetrahydro-1 H-3-benzazepine).*

Accumulating evidence over the past decade suggests that D2-like receptor agonists can prevent multiple pathological features found in AD. For instance, rotigotine, a DA_2/3_R agonist, could increase cortical excitability and restore central cholinergic transmission in patients with AD (Martorana et al., 2013) and reduce symptoms associated with frontal lobe cognitive dysfunction, thereby delaying impairment of activities of daily living (Koch et al., 2020). However, distinct ligands may allow D2-like receptors to exert a dual role in pathobiological activity of Aβ and tau. *In vitro*, both L-DOPA and piribedil promoted the generation of Aβ and increased the activity of γ-secretase, mediated by the activation of the DA_2_R and β2-arrestin signaling pathway in neuronal cells (Lu et al., 2017), whereas pretreatment with higher concentrations of DA_2_R agonist quinpirole protected neurons from Aβ toxicity (Melief et al., 2015). In addition, Koppel et al. (2016) reported that a tau mouse model of AD treated with DA_2_R antagonist haloperidol showed a significant reduction in tau phosphorylation associated with an inactivation of the tau kinase AMPK, whereas a study by Ziu et al. (2020) suggested an effective role of DA_2/3_R agonists in inhibiting tau aggregation. The underlying mechanism of this dual effect requires further verification.

### Physical Exercise, Dopaminergic System, and Alzheimer’s Disease

The effects of exercise on the dopaminergic system in PD patients and animal models has been widely reported (Hou et al., 2017; Sacheli et al., 2019). The dopaminergic system is also known to be involved in the effects of exercise on AD. Studies have shown that exercise can increase the contents of DA in the hippocampus. Using a microdialysis technique, Goekint et al. (2012) found that a bout of treadmill running for 60 min induced a twofold increase in hippocampal DA release in rats. In agreement with this, 30 min of treadmill running at 60–70% VO_2max_ rather than strength exercise ameliorated Aβ neurotoxicity by increasing hippocampal DA levels and promoted recognition learning in Aβ-induced rats (Rossi Dare et al., 2020). A chronic exercise intervention program consisting of 4 weeks of treadmill running (60 min/session, 5 sessions/week) before intraperitoneal LPS injection prevented LPS-induced loss of dopaminergic neurons in the SNpc, reduction in DA levels, and dysfunction of motor coordination (Wu et al., 2011). Furthermore, after swimming exercise for 4 weeks (30 min/session, 7 sessions/week), increases in the DA contents of the brain were found to be associated with improvements in learning and memory in AD rats induced by LPS, with the best effects for combined vitamin D and exercise treatment (Medhat et al., 2020). Thus, exercise appears to markedly improve LPS-induced cognitive and motor deficits by rescuing dysfunction of the dopaminergic system.

D1-like receptors are important participants in the improvements in cognition that occur under exercise stimuli. One study reported promotion of the persistence of object recognition memory and induction of the release of DA in the hippocampus as a result of 30 min of treadmill running at an intensity of 60–70% VO_2max_, whereas this effect was blocked by treatment with D1-like receptor antagonist SCH-23390 (Vargas et al., 2020). A recent study by Ramires Lima et al. (2021) identified specific mechanisms and found that a similar protocol activated D1-like receptors in rat hippocampus and improved memory persistence; however, the administration of SCH-23390 or inhibition of PKA but not PKC impaired the effect of acute aerobic exercise on memory persistence. In another study, 1 month of voluntary wheel running was shown to activate the DA_1_R/cAMP/PKA pathway, induce differentiation of hippocampal neurons, and enhance neurogenesis via the AMPK/CREB pathway in mice (Zang et al., 2017). Thus, signaling events mediated by PKA are critical for exercise to improve cognition. However, the effects of exercise on cognition mediated by D1-like receptors need further validation in models of AD in the future.

In AD brains, expression of D2-like receptors has been shown to be reduced in the cortex, striatal, and hippocampus regions (Pizzolato et al., 1996; Kemppainen et al., 2003; Kumar and Patel, 2007). Although research has proven that regular exercise can increase DA_2_R levels and improve dopaminergic signaling (Bauer et al., 2020), the relationships among exercise, DA, D2-like receptors, and cognition are not understood in sufficient detail. Several human studies offer some insight. In a cross-sectional study, the intensity of habitual physical activity of elderly individuals was found to be positively correlated with episodic memory and the availability of DA_2/3_R in the striatum, but the frequency of physical activity was not related to the availability of DA_2/3_R (Kohncke et al., 2018). In another study, elderly participants underwent an aerobic exercise intervention for 6 months, which led to significant increases in DA contents and improvements in working memory, compared with an active control; unexpectedly, DA_2_R levels decreased with exercise, and there was no relationship between DA_2_R and working memory at baseline or following exercise (Jonasson et al., 2019). Thus, the regulation of cognitive function by exercise through D2-like receptors is complicated, and it is still difficult to determine whether D2-like receptors are involved in the improvements in cognitive function of AD patients that are associated with exercise.

## Conclusion and Future Perspectives

**FIGURE 2:**
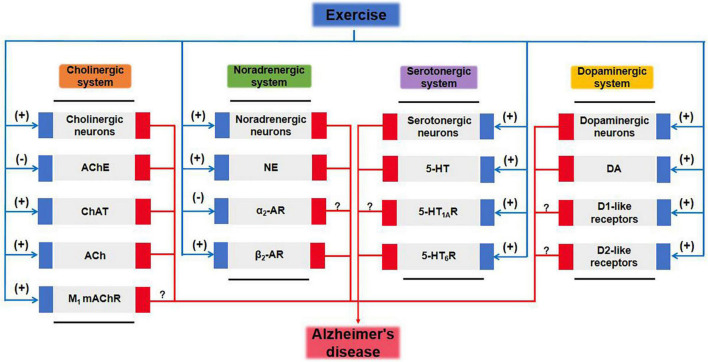
Cholinergic and monoaminergic systems mediate potential pathways for exercise amelioration in Alzheimer’s disease. 5-HT, 5-hydroxytryptamine; 5-HTR, 5-hydroxytryptamine receptor; ACh, acetylcholine; AChE, acetylcholinesterase; AR, adrenergic receptor; ChAT, choline acetyltransferase; DA, dopamine; mAChR, muscarinic acetylcholine receptor; MAOA, monoamine oxidase A; NE, norepinephrine.

Adverse changes in the cholinergic and monoaminergic systems of AD brains are mainly reflected in degeneration of cholinergic neurons and monoaminergic neurons; reductions in levels of neurotransmitters ACh, NE, 5-HT, and DA; and abnormalities of the activity of cholinergic receptors and monoaminergic receptors. Treatment with enzymes and proteins involved in the anabolism and catabolism of neurotransmitters and agents to target GPCRs can at least partially prevent multiple pathological features found in AD, including Aβ, tau, neurotoxicity, neuroinflammation, oxidative stress, synaptic dysfunction, and neuronal apoptosis. Further discussion of the relationship between neurotransmitters and distinct neurologic disorders including tauopathies would be very valuable to broaden the physiological functions of neurotransmitters and to explore therapeutic strategies. Traditional pharmacological therapies have failed to show long-term efficacy, and the specificity and possible side-effects of a pharmacological agent are always a concern. Fortunately, exercise therapy has significant promise as a highly efficacious, low-toxicity, and cost-effective therapy that can replace drugs to improve the function of the cholinergic and monoaminergic systems and enhance the cognitive performance of AD patients (Table 5 and Figure 2). Moreover, the effects of exercise combined with drug interventions are better than those of exercise interventions alone (Abhijit et al., 2017; Medhat et al., 2020; Shamsipour et al., 2021). However, in many exercise intervention studies, rodent models were selected as research subjects rather than humans, and the results reported in animals may not reflect what occurs in humans. In human intervention studies, combining biochemical and neuroimaging methods such as MRI, magnetic resonance spectroscopy, or positron emission tomography could provide fruitful avenues for research. Notably, the application of physical exercise-based interventions in humans has several limitations. Although such interventions are beneficial, patients with moderate-to-advanced disease usually experience limitations in their capacity for physical activity. For these patients, safety is a primary concern during the course of the exercise intervention; the elements of the intervention, including exercise type, intensity, frequency, and duration, need to be strictly controlled, which may require the supervision and guidance of a substantial number of specialized health care workers.

**TABLE 5:** Exercise effects on the cholinergic and monoaminergic systems in Alzheimer’s disease.

Subjects	Exercise type	Exercise intensity (Speed or other parameters)	Exercise duration	Mode of action	Relevance to AD	References
THY-Tau22 mice	Voluntary wheel running	ND	9 months	↑ Cholinergic neurons	↓ Tau and neuroinflammation ↑ Cognition	Belarbi et al., 2011
Aβ_1–40_-induced rats	Swimming (and/or donepezil hydrochloride)	Non-load	10∼60 min/session, 7 sessions/week for 4 weeks	↓ AChE ↑ ChAT	↓ Neuronal loss ↑ Cognition	Jiangbo and Liyun, 2018
Aβ_25–35_-induced rats	Treadmill running or climbing the ladder	10∼20 m/min 10∼100% BW	20∼40 min/session, 4 sessions/week for 8 weeks	↓ AChE	↑ Cognition	Farzi et al., 2019
Aβ_1–42_-induced rats	Treadmill running (and/or Probiotics)	10∼16 m/min	40∼85 min/session, 5 sessions/week for 8 weeks	↑ ACh	↓ Amyloid plaques and neuronal death ↑ Cognition	Shamsipour et al., 2021
Old Wistar rats	Voluntary wheel running	4∼15 rpm	5∼10 min/session, 5 sessions/week for 5 weeks	↑ NE, 5-HT, and DA	↓ Oxidative stress ↑ Cognition	Ramis et al., 2021
MCI patients	Stationary cycling	70% VO_2max_	6 min/session	↑ NE	↑ Cognition	Segal et al., 2012
Aged APP/PS1 mice	Treadmill running	10 m/min	20∼60 min/session, 5 sessions/week for 4 weeks	↑ Serotonergic neurons and cholinergic neurons	↓ Aβ_1–40_, Aβ_1–42_ and neuroinflammation ↑ Cognition	Ke et al., 2011
TgF344-AD rats	Treadmill running	18 m/min	45 min/session, 3 sessions/week for 8 months	↑ 5-HT and 5-HT_6_R	↓ Aβ, tau, oxidative stress and neuroinflammation ↑ Cognition	Wu et al., 2020
Aβ_25–35_-induced rats	Treadmill running	60∼70% VO_2max_	30 min/session	↑ DA	↑ Cognition	Rossi Dare et al., 2020
LPS-induced rats	Swimming (and/or vitamin D)	ND	30 min/session, 7 sessions/week for 4 weeks	↑ DA	↓ Aβ, tau, oxidative stress and neuroinflammation ↑ Cognition	Medhat et al., 2020

*5-HT, 5-hydroxytryptamine; Aβ, amyloid-β; ACh, acetylcholine; AChE, acetylcholinesterase; BW, body weight; DA, dopamine; LPS, lipopolysaccharide; MCI, mild cognitive impairment; ND, not determined; VO_2max_, maximum oxygen uptake.*

## Author Contributions

BZ prepared the first draft and final version of the manuscript. FY designed the diagrams and wrote the draft. XZ and WZ involved in literature searching. LL, SL, and PS critically edited and revised the manuscript. All authors contributed to the article and approved the submitted version.

## Conflict of Interest

The authors declare that the research was conducted in the absence of any commercial or financial relationships that could be construed as a potential conflict of interest.

## Publisher’s Note

All claims expressed in this article are solely those of the authors and do not necessarily represent those of their affiliated organizations, or those of the publisher, the editors and the reviewers. Any product that may be evaluated in this article, or claim that may be made by its manufacturer, is not guaranteed or endorsed by the publisher.
